# Neuromodulation guide for the non-neuromodulator clinician: What it is and how it can benefit patients?

**DOI:** 10.17305/bb.2024.10967

**Published:** 2024-08-11

**Authors:** Chelsey Hoffmann, Jinlan Wang, Rushna P Ali, Ryan S D’Souza

**Affiliations:** 1 Department of Anesthesiology and Perioperative Medicine, Mayo Clinic, Rochester, MN, USA; 2 Department of Spine, Mayo Clinic, Rochester, MN, USA; 3 Department of Neurosurgery, Mayo Clinic, Rochester, MN, USA

**Keywords:** Neuromodulation, stimulation, peripheral, deep brain, spinal cord

## Abstract

Neuromodulation is being utilized across a variety of medical subspecialties to treat both painful and non-painful medical conditions. However, publications on neuromodulation topics infrequently occur in journals targeting generalists and medical specialties outside of pain medicine and neurosurgery. This study reviewed implantable neuromodulation devices, their respective Food and Drug Administration (FDA)-approved indications for use, as well as off-label usage, and the associated potential risks and benefits for each device. PubMed and Medline databases were queried for systematic reviews with or without meta-analyses and randomized controlled trials of implantable neuromodulation devices. The literature review resulted in 106 studies eligible for inclusion, and 67 were included in the final review. In conclusion, as the clinical volume of neuromodulation continues to grow, supporting and educating medical professionals who care for patients who receive implanted neuromodulation devices is paramount. It is likely the use of neuromodulation will continue to expand across all medical subspecialties, and as such, every clinician should have a baseline understanding of this treatment.

## Introduction

Neuromodulation is utilized to treat a variety of conditions ranging from epilepsy to chronic pain. Its application spans the subspecialties of interventional pain management, neurosurgery, neurology, urology, gastroenterology, cardiology, vascular medicine, endocrinology, and more. While new publications highlight the usefulness of this therapy for specific subspecialty-related medical conditions, there remains a gap in neuromodulation knowledge and publications targeting general clinicians who do not offer neuromodulation as part of their standard clinical practice. For example, while several reviews have been published on the topic of non-invasive transcranial ultrasound neuromodulation for medical conditions, such as chronic pain, dementia, and epilepsy, this review differs by aiming to assist the reader in better understanding the definition and mechanisms of *implantable* neuromodulation. The review also aims to educate the reader on the various implantable neuromodulation anatomical targets, the indications approved by the Food and Drug Administration (FDA), as well as common off-label uses, and potential adverse events. To date, the authors of this manuscript are unaware of any existing broad reviews of implantable neuromodulation that provide guidance and education to generalists.

## Materials and methods

The PubMed and Medline databases were queried using the Population, Intervention, Comparator, and Outcomes (PICO) model. The population was defined as adults (age 18 years or older). The intervention of interest was *implantable* neuromodulation devices. The comparison was no intervention, conventional medical management, or sham stimulation. Outcomes of interest included improvements in pain relief and/or functional status. Search terms included neuromodulation, spinal cord stimulation (SCS), peripheral nerve stimulation (PNS), carotid sinus stimulation, deep brain stimulation (DBS), vagus nerve stimulation (VNS), and sacral nerve stimulation. Results were filtered by the following study types: meta-analyses, randomized controlled trials, and systematic reviews. Publications were included if published in the English language and with a publication date in the last ten years. Results were further restricted to those on the topics of *implantable* neuromodulation devices. Publications on external neuromodulation devices or intrathecal drug delivery systems were excluded.

## Results

**Figure 1.**
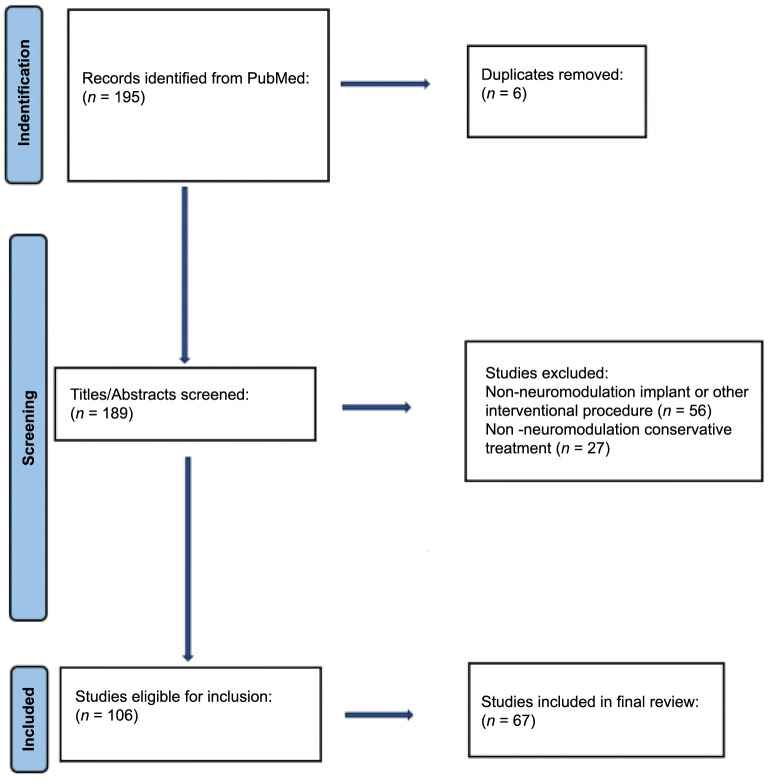
Flowchart of literature review strategy.

The initial literature search resulted in 195 publications. After duplicates were removed (*n* ═ 6), a manual review was performed on the remaining 189 articles. 83 articles were discarded due to the following reasons: non-neuromodulation implant or other interventional procedure (i.e., cardiac defibrillator, dental implant, intraocular implant, cardiac stent placement, cardiac ablation, or other [*n* ═ 56]), or non-neuromodulation conservative treatment (i.e., acupuncture or medication [*n* ═ 27]). The remaining 106 articles were screened for relevance to the purposes of this manuscript, and 67 were included in the final analysis. Figure 1 displays the article screening and selection process.

### Neuromodulation definition and general patient workup

Neuromodulation is defined by the International Neuromodulation Society (INS) as “the alteration of nerve activity through targeted delivery of a stimulus, such as electrical stimulation or chemical agents, to specific neurological sites in the body” [1]. Although electrical shocks from fish may have been utilized thousands of years ago for medical ailments, the modern-day use of electricity to treat various medical disorders began in the early 1960s [1].

While external neuromodulation devices (i.e., transcutaneous electrical nerve stimulation (TENS) or external neuromodulation for migraine headache) are available, the focus of this manuscript is on *implantable* neuromodulation devices. To that end, this manuscript will review currently available implantable neuromodulation devices, such as DBS, spinal cord stimulation (SCS), dorsal root ganglion (DRG) stimulation, PNS, sacral nerve stimulation, VNS, and carotid sinus stimulation.

**Figure 2.**
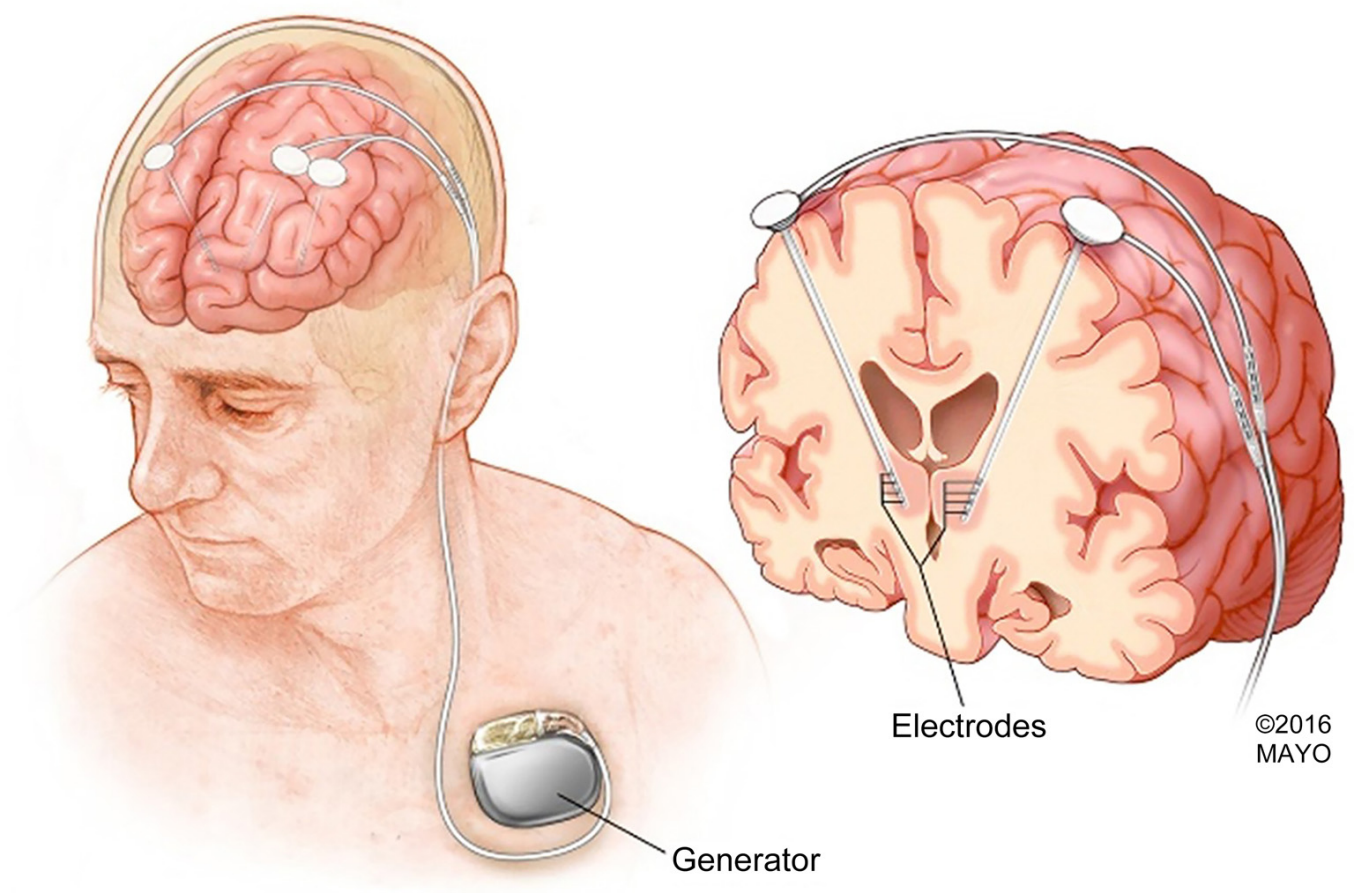
DBS with infraclavicular IPG Placement*. Intracranial electrodes/leads are tunneled to the IPG which is placed subcutaneously in the infraclavicular region. *Image reproduced with permission from Mayo Clinic. DBS: Deep brain stimulation; IPG: Internal pulse generator.

For all neuromodulation devices, patients require a baseline medical workup to ensure they are appropriate candidates for the therapy. Magnetic resonance imaging (MRI) should be obtained at the target implantation site to ensure there are no anatomical abnormalities that would pose barriers to the placement of the device (i.e., thoracic/lumbar MRI for SCS; brain MRI for DBS). Electromyography studies may also be useful to provide further information regarding target nerves of concern. Additionally, patients should be screened for the use of antiplatelet or anticoagulant medications, as these will need to be held for neuraxial or higher risk interventional procedures or bridged in patients who have a significant risk for clotting based on their risk factors [2]. Basic laboratory studies, such as a complete blood count, coagulation cascade (for those taking anticoagulants), and hemoglobin A1C (for diabetic patients) can be useful to ensure patients are not at an elevated risk of bleeding or infection. Additionally, many insurance carriers require patients to complete a psychological evaluation by a certified mental health professional, ideally prior to the trial of any neuromodulation device [3].

### Types of neuromodulation

#### Deep brain stimulation

DBS has been available for over 25 years [4]. The current FDA-approved indications for DBS include Parkinson’s disease, epilepsy, essential tremor, dystonia, and obsessive-compulsive disorder (OCD) [5].

DBS involves the implantation of electrodes within specific regions of the brain. The target region for electrode implantation depends upon the specific medical condition being treated. The electrical pulses produced by the implanted electrodes stimulate various nuclei and white matter tracts, impacting nerve firing and the release of various chemicals [6]. Commonly targeted nuclei for Parkinson’s disease include the Subthalamic Nucleus and Globus Pallidus Internus. Tremor responds to the Ventral Intermedius Nucleus of the Thalamus stimulation, whereas the Anterior Nucleus of the Thalamus (FDA-approved), Centromedian Nucleus (off-label), and Pulvinar (off-label) are utilized for medically refractory epilepsy.

The intracranial leads are implanted either with the patient awake, in conjunction with neuromonitoring and intraoperative testing, or asleep under general anesthesia utilizing intraoperative imaging such as intraoperative computed tomography (CT) or MRI. Various stereotactic frame-based and frameless techniques are used depending on the patient, disease, and institution-specific factors. The intracranial leads are then connected to extension wires which are tunneled to an internal pulse generator (IPG) and subsequently placed in the subcutaneous tissue in the infraclavicular region. Figure 2 illustrates a DBS and infraclavicular IPG placement.

In patients with Parkinson’s disease, DBS improves motor symptoms, predominantly tremor, rigidity, and akinesia, with some improvement in quality-of-life measures such as sleep [7, 8]. DBS reduces symptom severity and improves functional capacity and quality of life in adults with cervical, segmental, or generalized moderate-to-severe dystonia with a meaningful response in 50%–80% of those implanted [9]. Lastly, for patients with severe refractory OCD, DBS has been found to be a viable option for medically refractory cases [10].

Research on both DBS and brain-responsive neurostimulation (BRN) in patients with medically intractable epilepsy also continues to evolve. For example, a 2019 study followed 111 subjects with mesial temporal lobe epilepsy for 6.1 years ± (standard deviation) 2.2 years post-BRN implant and showed a median percent seizure reduction of 70% [11]. Additionally, a long-term follow-up study of patients implanted with deep brain anterior thalamus stimulation showed favorable efficacy and safety profiles with a 71% reduction in the most severe seizure type (focal to bilateral tonic-clonic) [12].

#### Spinal cord stimulation

SCS was initially introduced for reflex sympathetic dystrophy, now more commonly referred to as complex regional pain syndrome (CRPS). Subsequently, indications approved by the FDA have expanded to include persistent spinal pain syndrome type 2, non-surgical refractory low back pain (persistent spinal pain syndrome type 1), and painful diabetic neuropathy [13–16].

Before the implantation of a permanent SCS device, patients are first implanted with temporary leads into the dorsal column of the spine at the targeted spinal level. These temporary leads exit the skin and are connected to an external pulse generator (EPG). For a period of five to ten days (though occasionally extended), the patient will wear the device during normal daily activities and record their pain. At the end of the trial, the temporary leads are removed and it is determined whether the patient experienced substantial improvement in their pain and/or functional goals, usually defined by a threshold of 50% improvement or more. If this threshold is met, the patient may be a candidate to move forward with the implantation of a permanent SCS system. For patients with a prior thoracolumbar surgery, stenosis at the target level of implant, or a history of percutaneous lead migration, a buried trial of paddle leads may be indicated.

**Figure 3.**
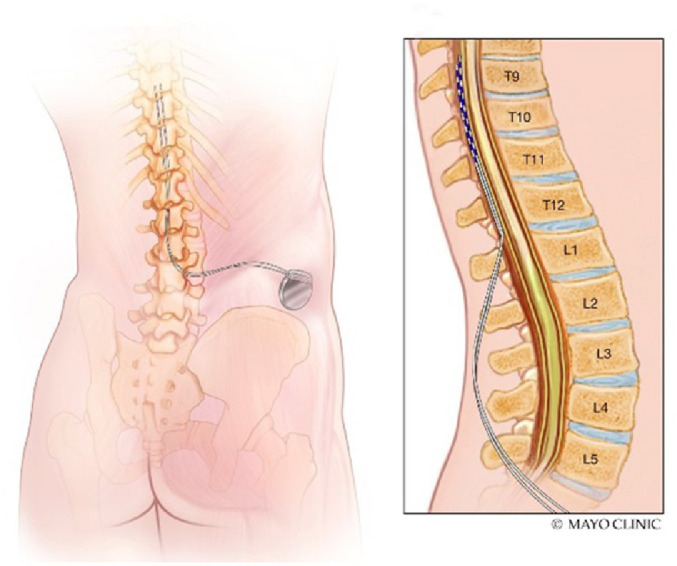
SCS with posterolateral flank IPG Placement*. Two spinal cord stimulator leads are placed in the dorsal column of the spine and subcutaneously tunneled to an IPG pocket in the posterolateral flank. *Image reproduced with permission from Mayo Clinic. IPG: Internal pulse generator; SCS: Spinal cord stimulation.

Permanent SCS implant involves the placement of two leads into the epidural space of the dorsal column at the target spinal level. The leads are then tunneled subcutaneously and connected to an IPG, which is placed in a pocket in the flank, buttock, chest wall, or other regions. Patients are followed closely in the post-operative period to ensure proper wound healing. Additionally, patients will have their SCS device programmed post-operatively and may require reprogramming throughout the lifespan of their device. Figure 3 illustrates an SCS system with posterolateral flank IPG placement.

Multiple waveforms, ranging from paresthesia-based to paresthesia-free, exist to provide patients with relief using SCS devices. Additionally, patients can utilize a remote control or smart phone-based application to operate their SCS system. For example, patients can turn the device on or off, increase or decrease the stimulation, and change between stimulation programs depending on their activities or personal preferences.

The most widely cited mechanism of action for SCS is the gate control theory, which was proposed by Melzack and Wall in 1965 [17]. The premise of the gate control theory is that SCS activates Aβ fibers within the dorsal column which, in turn, activate inhibitory interneurons in the dorsal horn of the spinal cord. The inhibitory interneurons then modulate incoming nociceptive input and release gamma-aminobutyric acid (GABA), an inhibitory neurotransmitter [18].

In a systematic review and meta-analysis by Hagedorn et al. (2022) [19] on patient satisfaction with SCS or DRG stimulation, 82.2% of participants were found to report satisfaction with their devices. Furthermore, another systematic review and meta-analysis by ElSaban et al. (2023) [20] showed a significant reduction in Oswestry Disability Index (ODI) scores up to 24 months post-implantation of an SCS device. At 12 months, pooled results revealed significant ODI improvement with a mean difference of −17.000% (95% CI: −23.07 to −10.94, *P* < 0.001) [20].

#### DRG stimulation

The DRG was identified as an additional spinal target for neuromodulation in the 1990s. The DRG is a collection of cell bodies of sensory neurons located on both sides of the spinal cord in the lateral epidural space [21]. It serves as part of the pain relay center between the periphery, spinal cord, and the central nervous system [21]. Researchers and interventional physicians have found the DRG to be an ideal target for capturing focal areas of pain, such as groin, knee, or foot pain. As such, DRG stimulation is currently approved by the FDA for the treatment of neuropathic pain from CRPS and/or causalgia in the groin and lower extremities [22]. DRG stimulation has not yet been approved for these conditions in the upper extremities.

Like SCS, patients can undergo a trial of DRG stimulation, whereby temporary leads are placed and connected to an EPG. Throughout the trial, the patient’s degree of pain and functioning are closely monitored. If the patient surpasses a defined threshold in improvement of pain and/or physical functioning, they may be a candidate to undergo permanent DRG system implantation. At the time of permanent implantation, the DRG leads are placed at the target spinal level and connected to an IPG, which is most commonly pocketed subcutaneously in the patient’s posterolateral flank. Again, like SCS, DRG stimulation patients are provided with a remote to operate their device and do require follow-up appointments to ensure proper wound healing and device programming.

Like other implantable neuromodulation devices, a variety of theories exist related to the proposed mechanism of action for DRG stimulation. These include, but are not limited to, inhibition of noxious and autonomic afferent signals and activation of the endogenous opioid system in the dorsal horn [23–27].

DRG stimulation has been well received by patients suffering from chronic intractable pain and has resulted in high levels of patient satisfaction [19]. Furthermore, research has shown as high as a 64% decrease in pain scores for patients suffering from CRPS Type I and a 58% decrease in pain for patients suffering from causalgia at 12 months post-DRG implant [21].

#### Sacral nerve stimulation

In the 1980s, sacral neuromodulation was developed to treat conditions such as overactive bladder, including the symptoms of urinary incontinence, urgency, or frequency [28]. Since 1997, sacral nerve stimulation has been FDA approved for use in the treatment of urinary incontinence and, beginning in 2011, for the indication of fecal incontinence [29, 30].

To ensure patient success with this therapy, patients undergo a temporary trial of the device while keeping a voiding diary. If the patient achieves 50% or greater improvement, they are then eligible to be considered for a permanent system implant [31]. For permanent implantation of a sacral nerve stimulator, leads are placed near the S2, S3, or S4 nerve roots, with subsequent connection to an IPG and implantation of the IPG in the gluteal region [28].

Currently, the proposed mechanism of action for sacral neuromodulation is that the therapy interferes with the afferent input to the sacral nerve roots of the spinal cord, inhibiting detrusor overactivity and resulting in clinical relief of urinary frequency and urgency [32].

Sacral nerve stimulation has been shown to be more effective in combination with antimuscarinic agents compared to antimuscarinic medication alone [33]. Additionally, several studies support the use of sacral nerve stimulation for urge incontinence or urinary retention [34, 35].

The FDA has also approved posterior tibial nerve stimulation (PTNS) for the treatment of overactive bladder and associated symptoms of urinary frequency, urgency, or urge incontinence [36]. PTNS involves the delivery of electrical current near the posterior tibial nerve at the ankle. This can be accomplished via a percutaneous needle electrode, a transcutaneous surface electrode, or more recently with the development of a wireless implantable tibial nerve stimulator [37]. The proposed mechanism of action for PTNS is that it increases suppression of the overactive detrusor muscle by increasing inhibitory spinal interneuron function [38].

In a systematic review and meta-analysis by Burton et al. (2014) [39], the pooled subjective success rate of percutaneous posterior tibial nerve stimulation for overactive bladder was 61.4% (95% CI: 57.5–71.8), and the objective success rate was 60.6% (95% CI: 49.2–74.7).

#### Vagus nerve stimulation

Implantable VNS studies occurred as early as the 1930s and 1940s [40]. Currently, VNS is FDA approved for the treatment of epilepsy, morbid obesity, treatment-resistant depression, and post-stroke rehabilitation [41]. Other medical conditions still undergoing research with VNS include, but are not limited to, rheumatoid arthritis-related inflammation, metabolic syndrome, disorders of consciousness, and heart disease.

There are now a variety of approaches to stimulating the vagus nerve, including a left or right cervical VNS implant as well as transcutaneous stimulation. In the most common approach, a lead is coiled around the left mid-cervical vagus nerve through an incision in the left neck region, which is then tunneled subcutaneously and attached to an IPG placed in the left upper chest [40]. Like the other neuromodulation devices described previously, an implanted VNS system requires post-implant programming visits, and patients are provided with a programmer to turn the system off. On-demand stimulation can be delivered by swiping a manufacturer provider’s magnet across the IPG.

The mechanism by which VNS benefits to patients with epilepsy is still unknown. However, there have been suggestions that the vagus nerve may impact regions susceptible to heightened excitability, such as the limbic system, thalamus, and thalamocortical projections [42]. VNS may also contribute to seizure suppression by affecting structures in the midbrain and hindbrain [43]. In terms of efficacy, 40% of patients using VNS have shown a 50% reduction in seizures after three years of treatment [44].

For depression, neuroimaging studies have suggested that VNS may result in mood-enhancing benefits by altering medial and prefrontal cortical transmission. These regions are responsible for releasing neurotransmitters, including serotonin and norepinephrine, which have both anticonvulsive and antidepressant effects [45]. In studies of patients with major depressive disorder who received VNS, 42% experienced a positive effect and 22% experienced remission after two years [46].

For obesity, a laparoscopically placed vagal nerve blockade device is available. This device emits intermittent electrical pulses to the intra-abdominal vagal trunks and has been shown to result in clinically significant weight loss [47]. Furthermore, a recent systematic review and meta-analysis demonstrated that vagal nerve therapy may result in mild-to-moderate weight loss over 12 months [48]. Stimulation of the vagus nerve is thought to induce weight loss by signaling stomach distension and producing satiety [49]. Though the exact mechanism continues to undergo additional research, vagus nerve blockade via neuromodulation may block aberrant signals that otherwise promote hyperphagia [50].

#### Peripheral nerve stimulation

PNS has experienced tremendous growth in recent years. This therapy can be utilized to treat acute and chronic painful neuropathic conditions existing in the periphery (i.e., outside of the brain or spinal cord). Current FDA-approved indications for PNS therapy include severe intractable chronic pain of peripheral nerve origin, post-surgical pain, and post-traumatic pain of the head, neck, trunk, and extremities [51–53]. While some PNS devices have been approved for use in the head and neck region, the implantation of some PNS systems in the craniofacial area could be considered off-label [53].

Modern-day PNS systems include both temporary and permanent devices. In each of the existing PNS systems on the market, a lead is placed near the target peripheral nerve and subsequently connected to either an IPG or EPG. PNS systems with lead connection to an IPG then involve the IPG being tunneled subcutaneously to a “pocket.” The device is then programmed so that electricity may modulate the ability of the nerve to “fire” or depolarize. For some PNS systems, a trial is available, whereby the patient has temporary leads implanted for five to seven days, or more. During this time, as with SCS trials, the patient documents their pain scores and ability to perform functional activities. If the patient achieves 50% or greater improvement in pain and functioning during the trial, they are then eligible to proceed to permanent implantation.

The current theory behind the mechanism of action for PNS includes both central and peripheral processes [51, 52]. PNS may reduce neurotransmitters and other inflammatory markers in the periphery while decreasing the transmission of pain. Centrally, PNS may attenuate wide dynamic range (WDR) neurons in the dorsal horn of the spinal cord, inhibit spinothalamic tract cells, and affect regional cerebral blood flow [51, 52].

**Table 1:** FDA approved indications for neuromodulation devices

**Device/Target**	**FDA approved indications**
Deep brain stimulation	Parkinson’s disease, epilepsy, essential tremor, dystonia, and obsessive-compulsive disorder [4, 5].
Spinal cord stimulation	Non-ischemic chest pain; chronic intractable pain of the low back, trunk, or limbs; and painful diabetic peripheral neuropathy of the lower extremities [13–16].
Dorsal root ganglion stimulation	Neuropathic pain from complex regional pain syndrome or non-surgical low back pain [22].
Sacral nerve stimulation	Urinary incontinence, urgency, or frequency [29, 30].
Vagus nerve stimulation	Epilepsy, depression, and post-stroke rehabilitation [41].
Peripheral nerve stimulation	Severe intractable chronic pain of peripheral nerve origin, post-surgical pain, and post-traumatic pain of the bank and/or extremities [51–53].
Carotid sinus stimulation	Heart failure – for patients who remain symptomatic despite treatment with guideline-directed medical therapy [56].*

*See other qualifiers as outlined within the manuscript under the subsection “*Carotid Sinus Stimulation*.” FDA: Food and Drug Administration.

According to a recently conducted systematic review of implantable PNS for peripheral neuropathic pain, low-quality evidence was found to support reduced pain intensity with PNS treatment for CRPS, shoulder pain, post-surgical pain, and mononeuropathies, whereas moderate-quality evidence was found for phantom limb pain [53]. Furthermore, the American Society of Pain and Neuroscience has published guidelines for the use of implantable PNS in the treatment of chronic pain [52]. These guidelines assign Level I, Grade B evidence for PNS treatment of the following conditions: chronic migraine headache, chronic hemiplegic shoulder pain, failed back surgery, lower extremity neuropathic pain, and lower extremity post-amputation pain.

#### Carotid sinus stimulation

Carotid sinus stimulation via implantable electrodes was first attempted in dogs in 1965 [54]. In 2004, the first clinical study evaluated the efficacy and safety of carotid baroreceptor stimulation in a human patient for the long-term treatment of hypertension [55]. Currently, the FDA-approved indication for this therapy is heart failure in patients who remain symptomatic despite treatment guideline-directed medical therapy, are New York Heart Association Class III or Class II (with a recent history of Class III), have a left ventricular ejection fraction ≤ 35%, and have an NT-proBNP < 1600 pg/mL, excluding patients indicated for Cardiac Resynchronization Therapy according to American Heart Association/American College of Cardiology/European Society of Cardiology guidelines [56].

Implantation of a carotid sinus baroreceptor stimulation system involves the placement of bilateral carotid sinus leads connected to an IPG. The IPG is pocketed in the upper chest wall region [57]. By stimulating the carotid sinus baroreceptors, impulses are transmitted to the brainstem and interpreted as elevated blood pressure. This results in the central nervous system modulating sympathetic and vagal outflows to reduce blood pressure and heart rate [57].

In research performed with subjects suffering from resistant hypertension who were treated with baroreflex activation therapy (electrical stimulation of carotid sinus baroreceptors), up to 76% of participants qualified as clinically significant responders, with a mean blood pressure decrease of 35/16 mm Hg [58]. Furthermore, long-term studies on carotid baroreceptor stimulation have shown reductions in left ventricular mass and posterior wall thickness [59].

### FDA-approved neuromodulation indications and off-label uses

A summary of FDA-approved indications for the previously described implantable neuromodulation devices and associated targets is displayed in Table 1. Off-label uses for implantable neuromodulation devices also exist. For instance, some off-label indications for dorsal column SCS include chemotherapy-induced peripheral neuropathy, sexual dysfunction, peripheral vascular disease, and refractory angina pectoris [60–62]. Off-label indications for DBS include central neuropathic pain, neurological recovery after stroke, traumatic brain injury, spinal cord injury, and psychiatric indications other than OCD.

### Risks of neuromodulation

General risks associated with any implantable neuromodulation device include bleeding, infection (superficial or deep), damage to nearby anatomical structures, misplacement of leads/electrodes, lack of benefit, pain, and other hardware-related complications (i.e., lead migration, lead fracture, battery malfunction, and programming problems) [63–66]. Furthermore, any neuromodulation implant procedure involving sedation will have risks associated with the use of anesthesia such as oversedation, airway compromise, nausea, and others. However, each neuromodulation device and its associated specific target result in more specific risks, outlined in Table 2.

**Table 2:** Risks of neuromodulation devices

**Device/Target**	**Risks**
Deep brain stimulation	Intracranial hemorrhage, brain abscess, erosion, stroke, and seizure [73, 74].*
Spinal cord stimulation	Epidural hematoma, nerve injury, dural puncture, and paralysis [75]. *
Dorsal root ganglion stimulation	Hematoma, neurological symptoms, and dural puncture [76].*
Sacral nerve stimulation	*
Vagus nerve stimulation	Cough, hoarseness, voice alteration, and paresthesias [77].*
Peripheral nerve stimulation	Skin erosion*
Carotid sinus stimulation	*

*Each of the above listed neuromodulation devices, as outlined within the manuscript under the section titled *Risks of Neuromodulation*, carry general risks associated with any implantable devices. These include, but are not limited to, procedural complications, serious adverse events, device-related complications, and patient complaints.

## Limitations

While the authors provided a comprehensive summary and overview of neuromodulation and its various indications for use, it is possible that additional neuromodulation targets and indications exist due to the evolving level of evidence. To minimize the risk of omitting neuromodulation targets or indications, the authors consulted the online resources available from the INS and North American Neuromodulation Society in addition to the research methods described in the methods section.

### Future directions

While this article primarily focuses on *implantable* neuromodulation devices, there are many neuromodulation devices on the market, which are worn by the patient or operate external to the human body. Therefore, a follow-up review of *external* neuromodulation devices, the associated FDA-approved indications and risks vs benefits is warranted. Additionally, non-invasive neuromodulation techniques, such as transcranial ultrasound, exist for a variety of medical conditions including pain, dementia, depression, epilepsy, and traumatic brain injury. Several reviews are available, that further elaborate on the FDA-approved indications and potential risks and benefits of this therapy [67, 68].

Given the increasing use of neuromodulation for off-label indications, further research is warranted to expand the existing evidence base and support FDA approval of these devices for patients who are suffering. For example, a 2022 systematic review on the efficacy of neuromodulation interventions for the treatment of sexual dysfunction found overall support for neuromodulation in improving sexual dysfunction [61]. Similarly, a 2022 review on patients with refractory angina pectoris concluded that SCS is a safe and effective treatment modality for patients suffering from this condition [62]. Lastly, a double-blind, crossover, placebo-controlled, single-center randomized controlled trial investigating the efficacy of SCS in patients with refractory angina pectoris is underway [62].

As neuromodulation devices and technology continue to advance, additional research is needed regarding patient outcomes associated with the size of the implanted neuromodulation device, the risks vs benefits and potential opportunities of remote programming, closed-loop technology, and the role of artificial interfaces to guide and personalize patient care [69–72].

## Conclusion

This article has defined neuromodulation, summarized the wide range of currently approved uses for implantable neuromodulation devices, discussed common off-label indications, and outlined the risks associated with these devices. As additional research arises, medical generalists will require updates to ensure that neuromodulation remains a part of the patient care treatment algorithm for a variety of medical conditions and disease states.
